# Low-Abundance *Dietzia* Inhabiting a Water-Flooding Oil Reservoir and the Application Potential for Oil Recovery

**DOI:** 10.1155/2019/2193453

**Published:** 2019-10-02

**Authors:** Peike Gao, Hongbo Wang, Guanxi Li, Ting Ma

**Affiliations:** ^1^College of Life Sciences, Qufu Normal University, Qufu, Shandong 273165, China; ^2^Research Institute of Experimental Detection, Xinjiang Oilfield Company, Petro China, Karamay, Xinjiang 834000, China; ^3^College of Life Sciences, Nankai University, Tianjin 300071, China

## Abstract

With the development of molecular ecology, increasing low-abundance microbial populations were detected in oil reservoirs. However, our knowledge about the oil recovery potential of these populations is lacking. In this study, the oil recovery potential of low-abundance *Dietzia* that accounts for less than 0.5% in microbial communities of a water-flooding oil reservoir was investigated. On the one hand, *Dietzia* sp. strain ZQ-4 was isolated from the water-flooding reservoir, and the oil recovery potential was evaluated from the perspective of metabolisms and oil-displacing test. On the other hand, the strain has alkane hydroxylase genes *alkB* and P450 CYP153 and can degrade hydrocarbons and produce surfactants. The core-flooding test indicated that displacing fluid with 2% ZQ-4 fermentation broth increased 18.82% oil displacement efficiency, and in situ fermentation of ZQ-4 increased 1.97% oil displacement efficiency. Furthermore, the responses of *Dietzia* in the reservoir accompanied by the nutrient stimulation process was investigated and showed that *Dietzia* in some oil production wells significantly increased in the initial phase of nutrient injection and sharply decreased along with the continuous nutrient injection. Overall, this study indicates that *Dietzia* sp. strain has application potential for enhancing oil recovery through an ex situ way, yet the ability of oil recovery in situ based on nutrient injection is limited.

## 1. Introduction

Oil reservoirs harbor diverse microbial populations that contribute to oil exploitation [1–3]. Among them, hydrocarbon-degrading bacteria are one of the most widely studied populations because of their important roles in the microbial enhanced oil recovery (MEOR) process. These microorganisms are generally able to produce surfactants with crude oil as a sole carbon source [4–6]. The produced surfactants can lower oil viscosity and oil-water interfacial tension to improve the recovery of residual oil underground. The products of oil oxidation, including fatty acids and surface-active agents, can serve as metabolic substrates for fermentative and methanogenic microorganisms that can produce fatty acids, alcohols, and gas. The metabolites improve oil recovery via reservoir repressurization, oil swelling, and decrease of oil viscosity [7].

Over the decades, many researchers devoted to isolate hydrocarbon-degrading bacteria from oil reservoirs and to investigate how these microorganisms improve oil recovery [5, 8–14]. To date, the isolated hydrocarbon-degrading microorganisms mainly belong to *Pseudomonas* [15–17], *Acinetobacter* [5], *Bacillus* [8, 18], *Aeribacillus pallidus* [19], *Bacillus licheniformis* [20], and *Geobacillus* [21]. These microbial populations were demonstrated to be able to enhance oil recovery in laboratory, and some species were found showing positive responses with incremental oil in laboratory and MEOR field trials [5, 8, 9, 22, 23].

With the development of molecular ecology, in particular, 16S rRNA high-throughput sequencing, more and more low-abundance microbial populations have been detected in oil reservoirs. Our previous investigation indicated that low-abundance *Dietzia* was generally detected in oil reservoirs, including 22 reservoirs with distinct different formation temperatures and salinity, across North, Northeast, and Northwest of China [24]. In addition, several *Dietzia* strains have been isolated from oil reservoirs and were found able to utilize hydrocarbons [25–27]. However, despite that, our knowledge about the oil recovery potential of these low-abundance microbial populations is poor. In this study, the distribution of *Dietzia* inhabiting a water-flooding oil reservoir was investigated. A *Dietzia* sp. strain ZQ-4 was isolated from the water-flooding reservoir, and the oil recovery potential was evaluated from the perspective of metabolisms and oil-displacing test. In addition, the responses of *Dietzia* in the reservoir accompanied by a nutrient stimulation process were investigated. The results will benefit our understanding about the roles of low-abundance *Dietzia* in the MEOR process.

## 2. Materials and Methods

### 2.1. Reservoir Information

The oil reservoir is located in Karamary Oilfield, Northwest China, has been subjected to water flooding since 2001. The temperature of the oil-bearing strata is 39°C, with a formation pressure of 14.71 MPa. The density of the crude oil is 0.862 g·cm^−3^, with a viscosity of 60.5 mPa·s. MEOR based on nutrient injection through water injection wells was performed in the reservoir. The nutrients consist of molasses, (NH_4_)_2_HPO_4_, and NaNO_3_. The production brines were collected from wellheads of the oil production wells through sampling valves and then completely filled in 15 L sterile plastic bottles and sealed with screw caps to avoid contamination and oxygen intrusion.

### 2.2. Microbial Community 16S rRNA Gene Sequencing

Total genomic DNA of microbial communities in the production brines was extracted using a bead shaker treatment and the AxyPrep™ Genomic DNA Miniprep Kit, Axygen, USA [22]. Universal primers 515f (GTG CCA GCM GCC GCG GTAA) and 806r (GGA CTA CHV GGG TWT CTA AT) were used to amplify the 16S rRNA V4 region (300–350 bp) according to the protocol described by Caporaso et al. [28, 29]. The PCR reaction mixture contained 10.5 *μ*L sterile ddH_2_O, 4 *μ*L 5× TransStart®FastPfu Buffer (TransGen Biotech, China), 2 *μ*L 2.5 mM dNTPs, 0.5 *μ*l TransStart®FastPfu DNA Polymerase, 0.5 *μ*L forward primer (10 *μ*M), 0.5 *μ*L reverse primer (10 *μ*M), and 2 *μ*L sample DNA. The reaction systems were denatured for 2 min at 95°C followed by 25 cycles at 94°C for 30 s, 50°C for 30 s, 72°C for 30 s, and a final elongation step at 72°C for 5 min. Amplicons were sequenced by pair-end sequencing on an Illumina MiSeq platform at Beijing Novogene Co. The raw sequences from sequencing were processed using Quantitative Insights into Microbial Ecology (QIIME) software package [30, 31]. Pairs of reads were merged using fast length adjustment of short reads (FLASH) [32]. The obtained sequences were demultiplexed and quality filtered using QIIME. The sequences were clustered into operational taxonomic units (OTUs) based on a threshold of 97% for sequence identity using UCLUST [33].

### 2.3. Isolation and Identification of *Dietzia* Strains

To isolate hydrocarbon-degrading *Dietzia* strains, 20 mL production brines were inoculated into 80 mL sterile basal salts medium (BSM) with 0.5% crude oil obtained from the oil production wells, and then, it was inoculated at 39°C and 180 rpm for 5 days. The BSM medium contained Na_2_HPO_4_ (0.6 g/L), KH_2_PO_4_ (0.2 g/L), NaNO_3_ (4 g/L), CaCl_2_ (0.01 g/L), FeSO_4_ (0.01 g/L), MgSO_4_ (0.3 g/L), and yeast extract (0.01 g/L), with pH equal to the production brines (7.5). Aliquots of 10^−3^ to 10^−5^ dilutions of the enrichment culture in which oil was emulsified were plated onto crude oil agar BSM and then incubated at 39°C until single colony formation.

The genome of the isolated strains was extracted with AxyPrep™ Genomic DNA Miniprep Kit (Axygen, USA). Microbial 16S rRNA genes were amplified using universal primers 27f (5′-AGA GTT TGA TCT GGC TCA G-3′) and 1492r (5′-TAC GGT TAC CTT GTT ACGACTT-3′) [34]. The obtained 16S rRNA amplicons were sequenced and compared with sequences deposited in the GenBank database to find the most closely related populations. Alkane monooxygenase (*alk*B) and cytochrome P450 (CYP 153) genes that are related to oil degradation were detected with degenerate primers. The alkBwf 5′-AAY CAN GCN CAY GAR CTN GGV CAY AA-3′ and alkBwr 5′-GCR TGR TGR TCH GAR TGN CGY TG-3′ were used to detect the *alk*B gene, producing approximately 550 bp amplicons [35, 36]. Primers P450F (5′-TGT CGG TTG AAA TGT TCA TYG CNM TGG AYC C-3′) and P450R (5′-TGC AGT TCG GCA AGG CGG TTD CCS RYR CAV CKR TG-3′) were used to amplify P450 genes, producing approximately 800 bp amplicons [35, 37]. Phylogenetic trees were constructed in MEGA 4 using the neighbor-joining method [38, 39]. The 16S rRNA gene and hydrocarbon-degrading gene sequences were deposited in GenBank databases under accession numbers JQ809512, MF188901, and MF188902, respectively.

### 2.4. Determination of Microbial Growth and Surfactant Production

The growth and surfactant production of the isolated *Dietzia* strain when growing on BSM with glucose, glycerol, bean oil, molasses, corn steep powder, and hydrocarbons as a sole carbon source were investigated. The growth curves were measured based on CFU (colony forming unit) counts: plating aliquots of samples on Luria–Bertani (LB) agar plates that were incubated at 39°C for 3 days. pH of the fermentation broths was measured by the pH test strip. Surface tension of the fermentation brines was measured using a digital tension meter (POWEREACH JK99B, China) at room temperature. The produced surfactants were separated from the culture medium by a solvent extraction method. After separation of biomass, the pH of the supernatant was adjusted to 2 with 6 M HCl and then extracted three times with an equal volume of chloroform/methanol (v/v, 2 : 1) in a separating funnel. The solvent layer was evaporated under vacuum to obtain extracts. The obtained extracts were ground with KBr powder and were dispersed uniformly in a matrix of paraffin for Fourier-transform infrared (FTIR) spectrometry measurement in the frequency range of 4,000–500 cm^−1^.

### 2.5. Determination of Oil Emulsification and Degradation

The oil emulsification and degradation ability of the isolated *Dietzia* strain were evaluated on BSM with 0.5% crude oil as a sole carbon source in aerobic, anoxic, and anaerobic conditions. Anoxic cultivation was accomplished by sealing an Erlenmeyer flask with a rubber stopper. Anaerobic cultivation was carried out in 300 mL serum bottles containing 200 mL medium and 0.1% resazurin. The serum bottles were then flushed with N_2_ flowed by sealing with a rubber stopper. The inoculated strain was harvested from precultures in the LB medium by centrifugation at 10, 000 ×*g* for 5 min and then washed twice with sterile BSM to exclude the residual organic compounds. Oil emulsion was evaluated based on the produced oil droplets using an optical microscopy with microscopic image analysis software, which yielded the average, maximum, and minimum diameters of the oil droplets. The residual crude oil was extracted with chloroform and then was separated into saturated hydrocarbons, aromatic hydrocarbons, asphaltene, and nonhydrocarbon fractions in a silica gel G column (60–120 mesh, 30 cm × 2 cm i.d.). The saturated hydrocarbons were analyzed by gas chromatography on Agilent 7890 equipped with the HP-5MS capillary columns (60 m × 0.25 mm i.d., 0.25 mm thickness).

### 2.6. Determination of Oil Displacement Efficiency in Cores

The oil recovery potential of isolated *Dietzia* strain was evaluated in rock cores. The core models were 29.5–29.8 cm in length and 2.1 cm in diameter, with pore percentages of 25.25–26.51% and water permeability of 0.517–0.601 *μ*m^2^. To evaluate the ex situ oil recovery potential of the isolated *Dietzia* strain, the oil-bearing cores were first water flooded until no oil was displaced out, and then, formation brines with 2% *Dietzia* sp. ZQ-4 fermentation liquor were injected into the core until no more oil was observed in the effluent. The control using the BSM medium containing 0.2% glycerol instead of fermentation liquor was performed in the same condition. To evaluate the in situ oil recovery potential of the isolated *Dietzia* strain, the oil-bearing core was first flooded by sterile formation brines with or without *Dietzia* sp. ZQ-4 (10^7^ cells/ml) until no oil was displaced out; then, 0.2 pore volume nutrient medium (0.6% NaNO_3_, 0.2% (NH_4_)_2_ HPO_4_, and 0.2% glycerol) prepared by production brines was injected into the cores. The cores were sealed and then incubated at 39°C for 7 days, followed by water flooding again until no further oil was obtained. Oil recovery efficiency and water content were calculated according to the volumes of displaced oil and displaced water in the core-flooding process.

## 3. Results and Discussion

### 3.1. Relative Abundances of *Dietzia* in the Oil Reservoir

**Figure 1:**
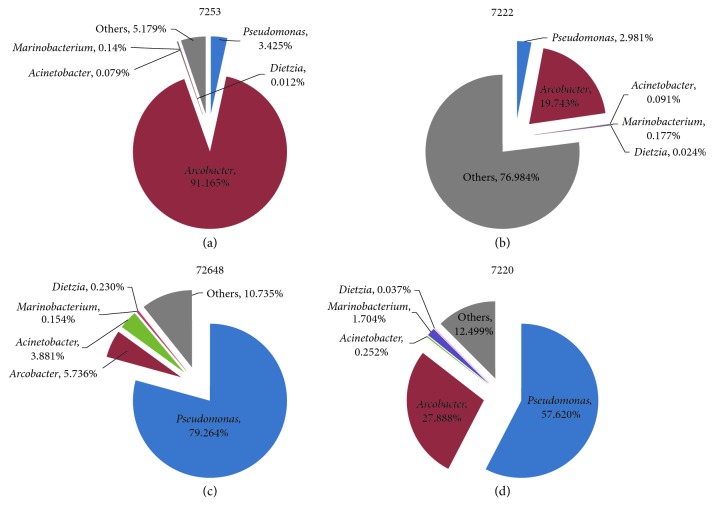
Distribution of dominant microbial populations and Dietzia in oil production wells (a) 7253, (b) 7222, (c) 72648, and (d) 7220.

Microbial communities in oil production wells of the oil reservoir were analyzed by 16S rRNA sequencing. Phylogenetic analysis showed that the dominant microbial populations belonged to *Proteobacteria*, *Firmicutes*, *Actinobacteria*, *Bacteroidetes*, *Thermotogae*, and *Synergistetes*. At the genus level, *Pseudomonas*, *Arcobacter*, *Acinetobacter*, and *Marinobacterium* were the most frequently detected populations (Figure 1). Although *Dietzia* is a kind of obligate aerobes, it was generally detected in the oil production wells, with its relative abundances of less than 0.5% (Figure 1). Our previous study also indicated that low-abundance *Dietzia* generally inhabited Chinese oil reservoirs [24]. Recently, Akbari et al. demonstrated that a nonmotile hydrocarbon-degrading *Dietzia maris* could migrate in submicrometer pores and low-permeability media [40]. In that case, *Dietzia* that inhabits oil reservoirs may be collected by flooding fluids from different habitats through oil-bearing strata.

### 3.2. Characterization of *Dietzia* sp. ZQ-4

**Figure 2:**
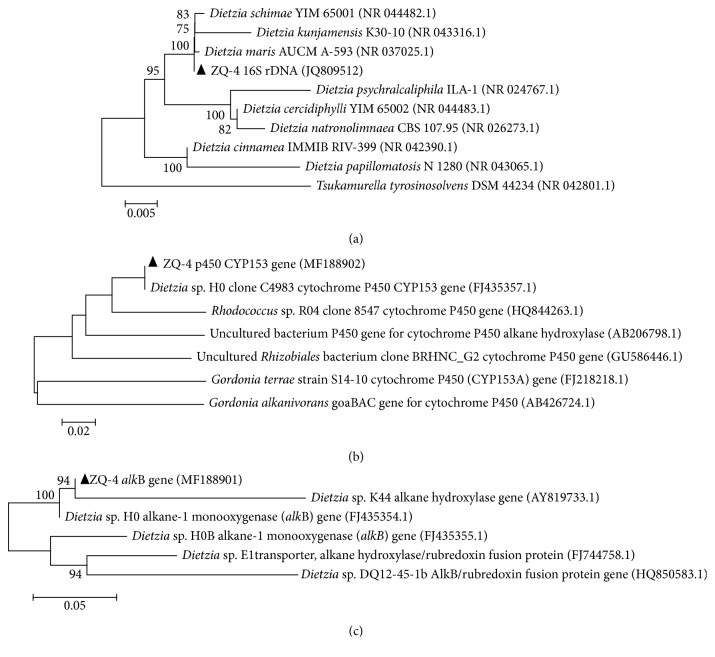
Phylogenetic relationships between Dietzia ZQ-4 and related species based on sequences of 16S rRNA gene (a), alkB gene (b), and CYP 153 gene (c).

Recently, several *Dietzia* strains have been isolated from oil reservoirs [25–27]. Despite the low abundance, *Dietzia* sp. ZQ-4 that could emulsify crude oil was isolated from the production brines. It showed 99% similarity of 16S rRNA gene with *Dietzia maris* gene (Figure 2(a)). Alkane monooxygenase *alk*B and cytochrome P450 (CYP 153) genes were detected in the strain (Figures 2(b) and 2(c)), indicating the potential for hydrocarbon degradation.

**Figure 3:**
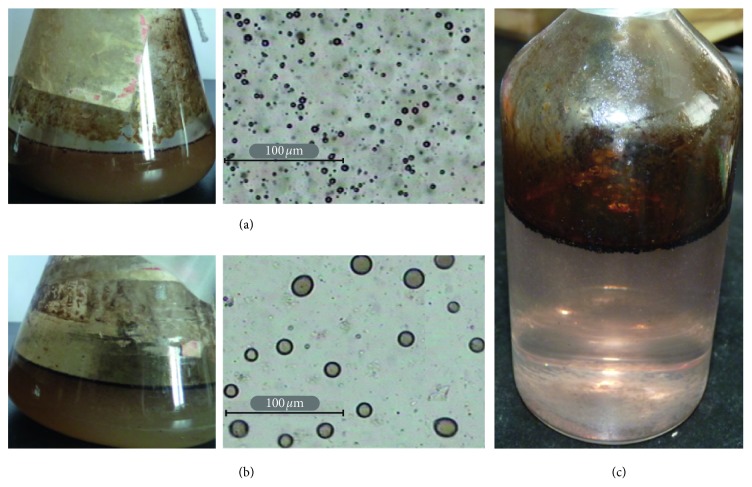
Oil emulsification by Dietzia ZQ-4 in aerobic condition (a), limited air supply condition with an air-liquid ratio of 2 : 1 (b), and anaerobic condition (c).

**Figure 4:**
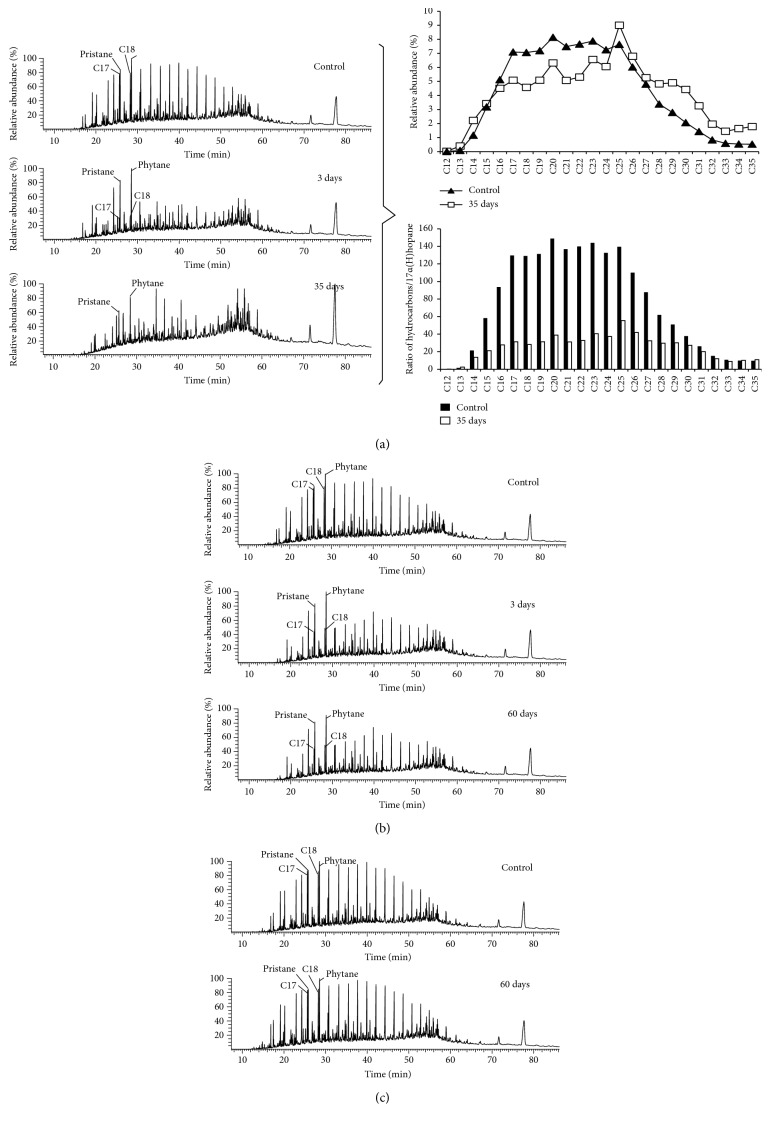
Gas chromatograms and relative proportions of different chain-length hydrocarbons in crude oil before and after Dietzia ZQ-4 treatment in aerobic condition (a), limited air supply condition (b), and anaerobic condition (c).

After ZQ-4 treatment in aerobic conditions, oil emulsion formed with an average oil droplet diameter of 4.7 *μ*m (97%< 10 *μ*m) (Figure 3(a)), saturated hydrocarbons of the crude oil decreased from 66.24 ± 0.74% to 55.64 ± 1.14%, and aromatic hydrocarbons increased from 14.50 ± 0.69% to 18.65 ± 0.53% (Table 1). GC analysis showed that ZQ-4 preferentially degraded *n*-alkanes ranging from C_14_ to C_33_ (Figure 4(a)). There were also research studies showing that *Dietzia* strains with *alk*B and CYP 153 genes could utilize C_6_ to C_40_ [26, 41, 42]. Microemulsions formed with an average oil droplet diameter of 12 *μ*m (97% < 23 *μ*m) after ZQ-4 treatment in anoxic conditions (V_liquid_/V_air_ = 1/2) (Figure 3(b)), and the saturated hydrocarbon decreased from 65.80 ± 1.01% to 56.96 ± 0.81% (Table 1). Oil degradation and emulsification were not obviously observed in anaerobic conditions (Figure 4(c) and Table 1). Although a previous study reported that *Dietzia maris* CBMAI 705 was able to degrade phenanthrene and methylphenanthrenes [43], aromatic hydrocarbon degradation by ZQ-4 were not obviously observed (Table 1). The hydrocarbon-degrading ability makes *Dietzia* be able to survive with crude oil as a sole carbon source in oil reservoir environments.

**Figure 5:**
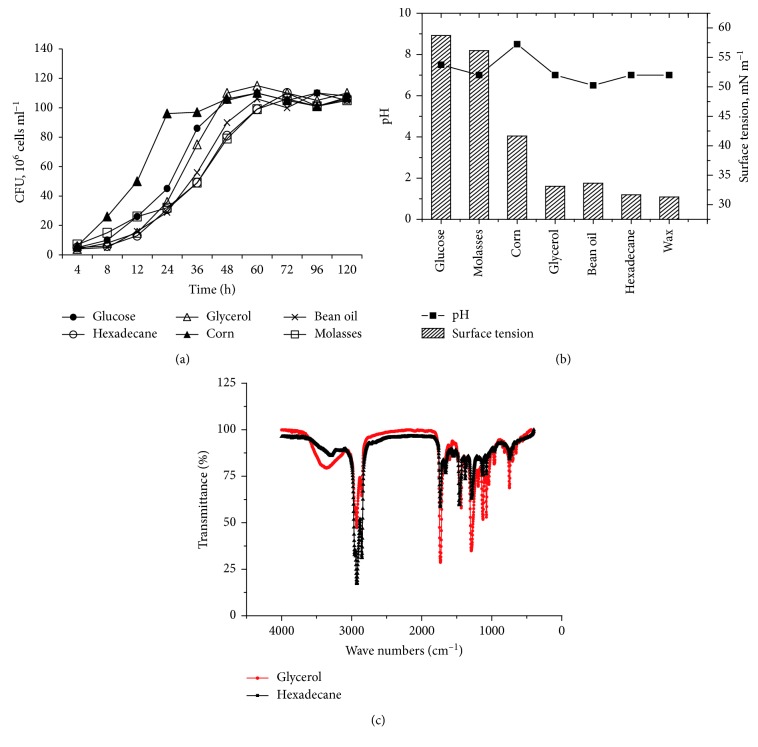
Growth of Dietzia ZQ-4 on BSM with different carbon sources as a sole carbon source (a), surface tensions of the fermentation broths (b), and infrared spectrogram of the produced surfactants with glycerol and *n*-hexadecane as a sole carbon source (c).

Biosurfactants were considered to be a critical role in a MEOR process. Biosurfactants can effectively improve the sweep efficiency of oil-bearing rocks to recover the residual oil underground through decreasing oil viscosity and interfacial tension of oil-water [2, 3, 44]. Strain ZQ-4 was found to be able to produce surfactants in BSM with glycerol, corn steep powder, vegetable oil, and hydrocarbons as a carbon source, respectively (Figure 5). As a result of the surfactant production, the surface tension of the culture was reduced to 32 mN/m. Infrared spectrogram showed that the produced surfactant with glycerol and *n*-hexadecane as a sole carbon source had the NH group (3330 cm^−1^), CH_3_ group (2925 cm^−1^ and 1434 cm^−1^), CH_2_ group (2850 cm^−1^ and 1380 cm^−1^), CONH_2_ group (1548 cm^−1^), CN group (1548 cm^−1^), and lactone group (1171 cm^−1^ and 1731 cm^−1^), indicating that the produced surfactant contains lipopeptides (Figure 5(c)).

### 3.3. Oil Displacement Efficiency of *Dietzia* sp. ZQ-4 in Cores

**Figure 6:**
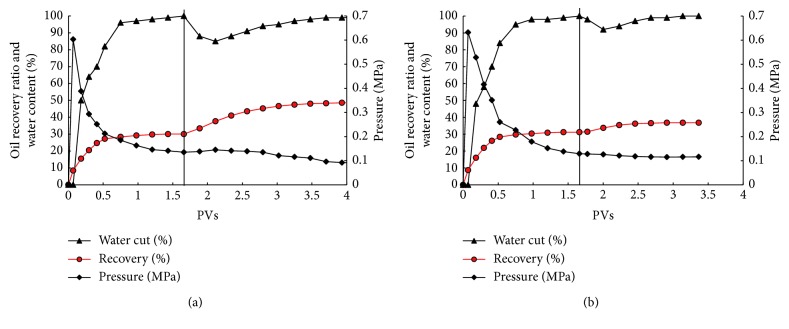
The water ratio, oil recovery ratio, and injection pressure during core-flooding tests: (a) ex situ oil recovery potential of Dietzia sp. ZQ-4 in core test; (b) in situ oil recovery potential of ZQ-4 in the core test accompanied by a nutrient stimulation process. The vertical bars in (a) and (b) indicate the injection of formation brines with fermentation liquor of ZQ-4 and nutrient injection, respectively.

Figure 6(a) shows the oil recovery and water ratio in the core-flooding process and revealed the oil displacement efficiency of ZQ-4 fermentation liquor. A large number of research studies have reported the oil recovery potential of *Bacillus subtilis*, *Bacillus licheniformis*, *Pseudomonas aeruginosa*, *Rhodococcus erythropolis*, and *Thermoanaerobacter* [18, 45–49]. These strains and their metabolites were found to be able to enhance oil recovery by 4.89–31% in the core-flooding test. In this study, displacing fluid with 2% ZQ-4 fermentation broth could significantly increase oil displacement efficiency (18.82%) in cores, showing the oil recovery potential. The in situ fermentation of ZQ-4 in cores was also performed to evaluate the in situ oil displacement efficiency of ZQ-4. As shown in Figure 6(b), ZQ-4 in situ fermentation increased 1.97% oil displacement efficiency. The results indicate that ZQ-4 has the application potential in MEOR through an ex situ way, yet it is hard to improve oil recovery in situ based on nutrient solution injection. Combined with the above results, the limited dissolved oxygen in injected aerated nutrient solution is apparently the crucial constraint, which determines whether ZQ-4 can massively grow and produce sufficient surfactants to improve oil recovery in the hypoxic environment.

### 3.4. Responses of *Dietzia* in the Reservoir in the Nutrient Injection Process

**Figure 7:**
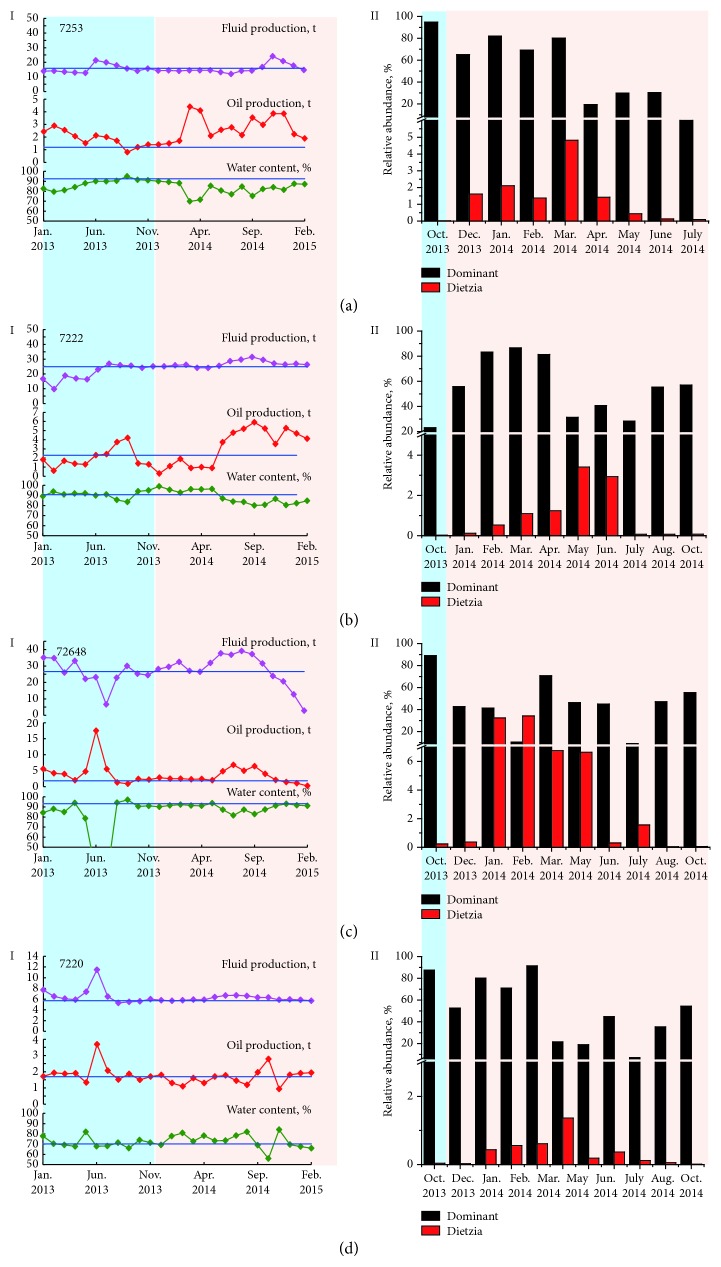
Oil production performances (I) and changes of dominant microbial populations (II) in the oil production wells 7253 (a), 7222 (b), 72648 (c), and 7220 (d) in the nutrient injection process. In part II, the dominant populations refer to *Pseudomonas, Arcobacter, Acinetobacter*, and *Marinobacterium*.

Community analysis showed that *Pseudomonas*, *Arcobacter*, *Acinetobacter*, and *Dietzia* significantly increased in the oil production wells when aerated nutrient solution was injected into the reservoir trough injection wells (Figure 7), indicating that *Dietzia* grew in oil-bearing strata. Our previous MEOR field trial in a low-temperature reservoir has also found that some undetected microbial populations, such as *Dietzia*, *Shewanella*, *Rhizobium*, and *Rhodococcus*, clearly increased during the nutrient injection process, yet the relative abundances of these microbial populations were less than 1% in the communities at a concentration of 10^7^−10^8^ cells mL^−1^ [22]. According to the production curves, the oil production wells were classified into two groups. For production wells 7253 and 7222, the liquid yield did not obviously change, whereas the oil production increased and water content decreased (Figures 7(a) and 7(b)). For production wells 72648 and 7220, oil increment and water content did not show obvious differences during the nutrient injection process (Figures 7(c) and 7(d)). Although incremental oil was obtained from production wells 7253 and 7222, it is hard to assess the contributions of the increased microbial populations, in particular *Dietzia*. For instance, *Pseudomonas*, *Arcobacter*, *Acinetobacter*, and *Dietzia* predominated production wells 72648 and 7220 during the nutrient injection process, yet there was no obvious oil increment (Figures 7(c) and 7(d)). In addition, with the continuous nutrient injection, *Dietzia* significantly decreased in the above oil production wells, in which *Pseudomonas*, *Arcobacter*, and *Acinetobacter* still predominated.

## 4. Conclusions

This study investigated the ex situ and in situ oil recovery potential of low-abundance *Dietzia* that inhabits a water-flooding oil reservoir. A *Dietzia* sp. ZQ-4 with the ability of hydrocarbon degradation and surfactant production was isolated, and the oil recovery potential was evaluated from the perspective of metabolisms and oil-displacing test. The results showed that displacing fluid with 2% ZQ-4 fermentation broth could significantly increase oil displacement efficiency in the core-flooding test, whereas in situ fermentation of ZQ-4 increased 1.97% oil displacement efficiency. Field trials with nutrient injection showed that *Dietzia* significantly increased in some oil production wells after nutrient injection yet sharply decreased along with continuous nutrient injection. Collectively, the results benefit our understanding about the roles of low-abundance *Dietzia* in the MEOR process: *Dietzia* can enhance oil recovery through an ex situ way and is limited to in situ oil recovery based on nutrient injection.

## Figures and Tables

**Table 1:** Relative contents of saturated hydrocarbon and aromatic hydrocarbon in crude oil before and after ZQ-4 treatment.

Experiment groups	Saturated hydrocarbon (%)	Aromatic hydrocarbon (%)
Oil degradation in aerobic condition		
Control	66.24 ± 0.74	14.50 ± 0.69
3 days	55.64 ± 1.14	18.65 ± 0.53
Oil degradation in limited oxygen supply condition (V_air_/V_liquid_ 2 : 1)		
Control	65.80 ± 1.01	13.05 ± 1.01
60 days	56.96 ± 0.81	16.72 ± 0.61
Oil degradation in anaerobic condition		
Control	65.39 ± 1.01	16.76 ± 1.02
60 days	64.84 ± 0.73	16.88 ± 0.26

## Data Availability

The data used to support the findings of this study are included within the article. The original data related to this study are available from the corresponding author upon request.
